# Landscape and dynamics of TadA-dependent RNA editing in *Escherichia coli* reveal a role in nutrient-rich growth

**DOI:** 10.1128/mbio.00551-26

**Published:** 2026-04-03

**Authors:** Danielle Arad, Ofir Fargeon, Liron Levin, Sine Lo Svenningsen, Liam Aspit, Dan Bar-Yaacov

**Affiliations:** 1 The Shraga Segal Department of Microbiology, Immunology, and Genetics, Ben-Gurion University of the Negev, Be'er-Sheva, Israel; 2 Bioinformatics Core Facility, Ben-Gurion University of the Negev, llse Katz Institute for Nanoscale Science and Technology 26732 , Be'er-Sheva, Israel; 3 Department of Biology, University of Copenhagen 56603, Copenhagen, Denmark; National Institutes of Health, Bethesda, Maryland, USA

**Keywords:** RNA editing, gram-negative bacteria, TadA, tRNA modification

## Abstract

Adenosine-to-inosine (A-to-I) mRNA editing alters genetic information post-transcriptionally and can impact protein sequence and function, yet its regulation in bacteria remains unclear. Here, we profiled A-to-I editing in *Escherichia coli* across nutrient-rich Luria-Bertani (LB) and minimal M9 media and different growth phases. Our analysis expanded the repertoire of TadA-dependent A-to-I edited mRNAs to 27, including 12 novel sites, and revealed that editing levels were dynamic and markedly increased at the stationary phase in LB but not in M9. Editing levels were independent of mRNA expression yet correlated with tRNA-Arg2 downregulation, and overexpressing tRNA-Arg2 reduced mRNA editing, demonstrating substrate competition for TadA, the sole bacterial tRNA adenosine deaminase. Mutants with TadA-deficient editing or reduced tRNA-Arg2 expression displayed similar LB-specific growth defects. Moreover, tRNA-Arg2 expression, tRNA-Arg2-dependent codon usage, and tRNA-Arg2 editing were all elevated in LB compared to M9. These findings establish regulatory principles for bacterial RNA editing, implicate tRNA editing in nutrient-responsive fitness, and provide a framework to explore the physiological roles of mRNA editing.

**IMPORTANCE:**

Adenosine-to-inosine (A-to-I) mRNA editing is a recently discovered post-transcriptional mechanism in bacteria, yet its regulation and physiological roles remain poorly understood. In this study, we expand the catalog of TadA-dependent mRNA editing events in *Escherichia coli* and identify key regulatory factors influencing editing levels, including nutrient availability, growth phase, and tRNA-Arg2 expression. Linking altered tRNA-Arg2 levels and editing to growth defects specifically in rich medium further demonstrates that tRNA editing contributes to nutrient-responsive fitness. Together, these findings establish a framework to explore bacterial RNA editing as a regulated process with potential implications for environmental adaptation and cellular function.

## INTRODUCTION

Adenosine-to-inosine (A-to-I) mRNA editing is a post-transcriptional modification that alters the genetic information encoded in mRNAs (1). This process is catalyzed by adenosine deaminases, which convert adenosine (A) to inosine (I) through deamination (1). In eukaryotes, A-to-I RNA editing is mediated by the ADAR (adenosine deaminase acting on RNA) family of enzymes and has been shown to be important for various cellular processes and organismal health; among others, it is essential for preventing aberrant double-stranded RNA immune responses, is crucial for proper neuronal activity, has been suggested to play a role in cancer progression, affects embryonic development, and diversifies the proteome (1–21). In bacteria, A-to-I RNA editing is facilitated by TadA (tRNA adenosine deaminase), an enzyme identified initially for its role in modifying tRNA-Arg2 at the wobble position (adenosine 34—A34) (22, 23). However, we previously showed that TadA also mediates mRNA editing in bacteria (in *Escherichia coli*) (23). Nevertheless, the governing principles of A-to-I mRNA editing in bacteria are poorly understood.

The discovery of A-to-I mRNA editing in *Escherichia coli* and other bacterial species has challenged the long-standing assumption that bacterial RNA editing was limited to tRNAs (23). Unlike ADARs in metazoa, which specifically target double-stranded RNA regions in mRNAs, bacterial TadA appears to recognize a specific sequence motif (UACG) and RNA secondary structure reminiscent of the tRNA-Arg2 anticodon stem-loop region (23–25). Recently, TadA homologs together with additional proteins (the Tad2–Tad3–Ame1 complex) were shown to mediate mRNA editing in fungi, representing a second A-to-I mRNA editing system distinct from animal ADARs (26). Similar to bacteria, the Tad2–Tad3–Ame1 complex preferentially recognizes local RNA secondary structures, supporting evolutionary conservation in the structural requirements of TadA and its homologs for editing (22, 23, 26).

What other factors affect or regulate A-to-I mRNA editing in bacteria? Previously, we observed that editing in the transcript of *hokB* was elevated as bacterial density increased in liquid culture (LB) (23). In contrast, no effect of growth phase was identified in the pathogenic gram-positive bacterium *Streptococcus pyogenes* (24). Moreover, A-to-I mRNA editing levels decreased in the *badR* transcript of the gram-negative bacterium *Klebsiella pneumoniae* in the stationary phase (25). These differences could represent a species-specific or a gene-specific regulatory program that responds to changes in nutrients throughout culture growth. Thus, editing levels in all transcripts should be examined in different species across growth phases. However, in *E. coli*, editing levels were never examined as a function of growth phases and nutrient availability.

Another factor that can affect A-to-I mRNA editing is the expression of TadA. Previously, we showed that overexpressing TadA in *E. coli* increased the amount of edited mRNAs from 15 to more than 300 (23). Thus, in *E. coli*, changes in TadA expression could increase the editing level and the repertoire of edited mRNAs (23). As noted, TadA edits both tRNA-Arg2 and multiple mRNAs. Thus, competition between tRNA-Arg2 and different mRNAs for available TadA units exists. Notably, the abundance of tRNA-Arg2 within individual *E. coli* cells was estimated at 4,752 (±440) (27). In contrast, the estimated average number of mRNA molecules per gene per *E. coli* cell is 0.4–3 (28, 29). Thus, tRNA-Arg2 abundance is at least two orders of magnitude higher than the combined molecule number of previously identified edited mRNAs. Furthermore, newly synthesized unedited tRNA-Arg2 levels drop at the stationary phase (30). Therefore, tRNA-Arg2 levels may affect A-to-I mRNA editing in *E. coli*.

Here, we explore the landscape of A-to-I mRNA editing in *E. coli*, focusing on its regulation across different growth phases, media conditions, and the interplay between TadA’s substrates. Our work provides insights into the regulatory principles underlying bacterial RNA editing and lays the groundwork for future research into its functional significance.

## RESULTS

### The landscape of A-to-I RNA editing in rich and minimal media across growth phases in liquid culture

**Fig 1:**
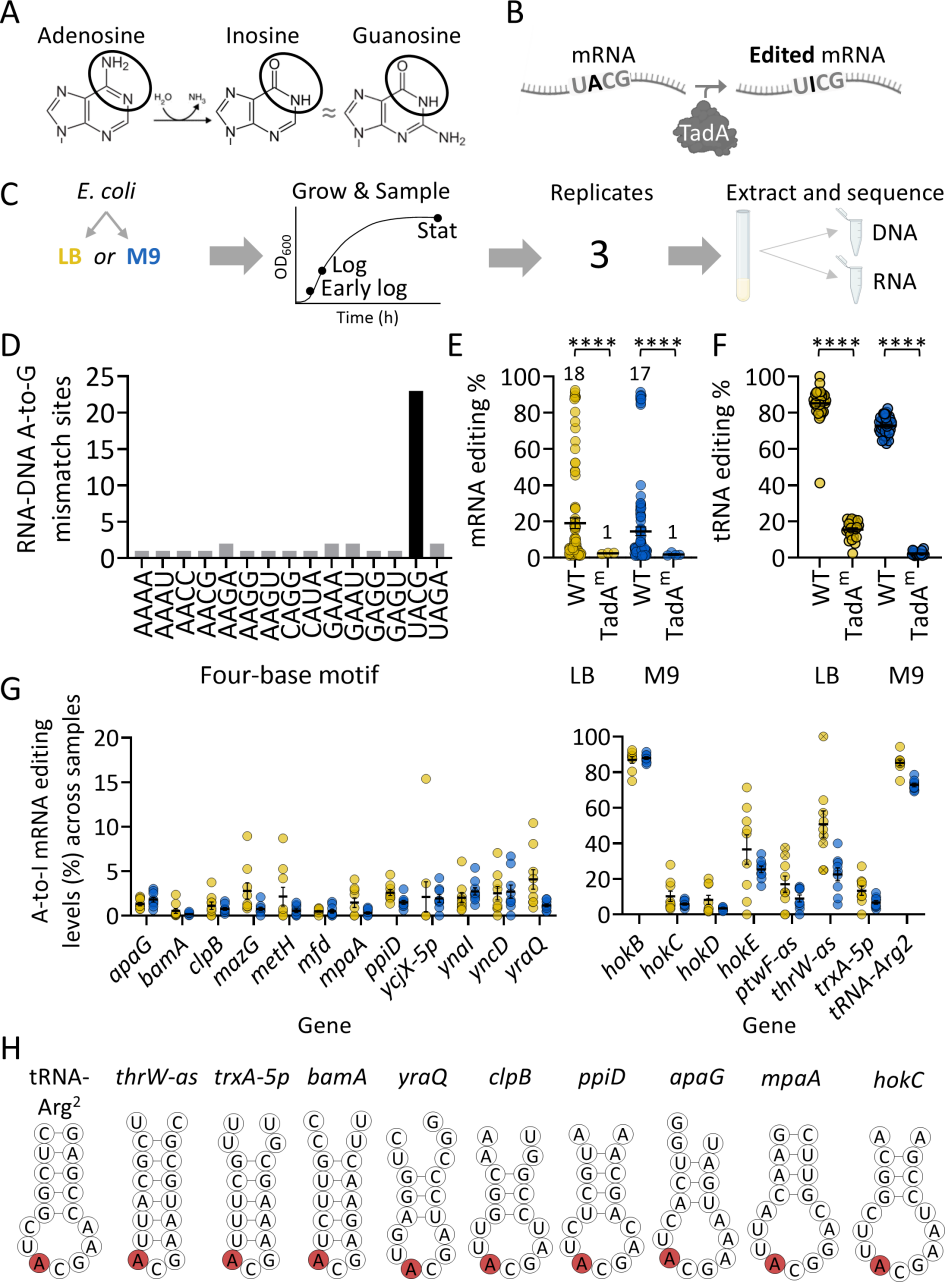
The landscape of A-to-I RNA editing in rich and minimal media across growth phases in liquid culture. (**A**) Adenosine is deaminated to inosine, which is recognized as guanosine by the reverse transcriptase, allowing its detection in sequencing data. (**B**) TadA can edit mRNAs containing a four-base sequence motif of UACG (TACG at the DNA level). (**C**) Experimental design: WT and TadA^m^ strains (23) of *E. coli* were grown at 34°C in either LB or M9. RNA and DNA were extracted from the same sample at three time points. Each sample was collected in three biological replicates (we repeated the entire experiment three times on different days). (**D**) Sequence motif distribution around A-to-G RNA-DNA mismatches in their genomic context. (**E**) A-to-I mRNA editing levels in the WT and TadA^m^ strains in LB and M9. Each data point represents a single editing event from all analyzed samples of each group. The numbers at the top of the graph denote the observed number of editing sites that pass our filters. (**F**) A-to-I tRNA editing levels in the WT and TadA^m^ strains in LB and M9. Each data point represents a single editing event. Statistical analysis in panels E and F was conducted with an unpaired Student’s *t*-test: *P* value ≤ 0.0001 (****). (**G**) An overview of RNA editing levels in identified transcripts. The left panel shows sites with an average editing of below 20%. The right panel shows sites with an average editing of above 20%. Yellow, LB; blue, M9; each data point represents a single editing event, and data points marked with “x” have read coverage lower than 10 reads. (**H**) Minimum free energy secondary structure predicted by RNAfold (31) around the A-to-I editing site (red) for the 17 nucleotides composing the anticodon arm of tRNA-Arg2 and for the 17 nucleotides around the edited site in 10 representative mRNAs. Predicted structure of all edited mRNAs is found in Fig. S3 and S4.

First, we aimed to determine the A-to-I mRNA editing landscape in rich (LB) and minimal (M9) media across growth phases. To this end, we sequenced RNA and DNA from *E. coli* to identify A-to-G RNA-DNA mismatches with frequency ≥1% (Fig. 1A through C; Fig. S1). Importantly, we used the WT strain and a *tadA*-mutant strain (TadA^m^) carrying a point mutation in the endogenous *tadA* locus, resulting in an aspartic to glutamic acid substitution (D64E) located four residues upstream of the catalytic HAE motif in the active site, which affects TadA activity *in vitro* and *in vivo* (22, 23). Examining the sequence around the A-to-G mismatches revealed that they are significantly enriched in a UACG sequence motif (Fig. 1D; Fig. S1, S2A and B; Tables S1 to S3). Focusing on the UACG putative sites revealed that editing levels significantly decreased across all sites in the TadA^m^ compared to the WT strain (Fig. 1E and F). Notably, editing in tRNA-Arg2 was almost completely abolished in the TadA^m^ when cultured in M9 (Fig. 1F). This is surprising because editing was thought to be essential in bacteria for decoding multiple arginine codons by tRNA-Arg2. In contrast to the UACG-embedded sites, no significant change in variant levels was observed in the non-UACG sites between the WT and TadA^m^ (Fig. S2C). Thus, the UACG-embedded sites represent bona fide TadA-dependent A-to-I editing events.

In total, we identified 19 TadA-dependent A-to-I mRNA editing sites in addition to the canonical tRNA-Arg2 edited site (Fig. 1G). Importantly, we validated six mRNA editing events using Sanger sequencing, showing they only occur at RNA but not in DNA samples (Fig. S2D). Interestingly, the one site that could not be validated (*ptwF-as*) resides in an antisense RNA of a known gene (*ptwF*). Thus, it is possible that expression from the sense RNA (*ptwF*) hinders validation by Sanger sequencing, as the PCR will amplify cDNA from both RNAs.

Finally, as previously shown in the case of *hokB*, most mRNA editing events (15/19) are predicted to be embedded in a stem-loop structure reminiscent of the anticodon arm of tRNA-Arg2 (Fig. 1H; Fig. S3 and S4). Importantly, the stem-loop structure was shown to be required for TadA-dependent tRNA-Arg2 editing (22). Among the 19 sites, 10 are novel and were not reported to occur endogenously. Importantly, 8/10 novel sites were identified previously upon overexpression of TadA (23). Furthermore, six previously identified sites were not identified in the present analysis (23). Thus, our analysis expanded the repertoire of TadA-dependent editing sites and revealed that a subset of mRNA editing events could vary between experiments.

### Growth medium and phase affect A-to-I RNA editing levels

**Fig 2:**
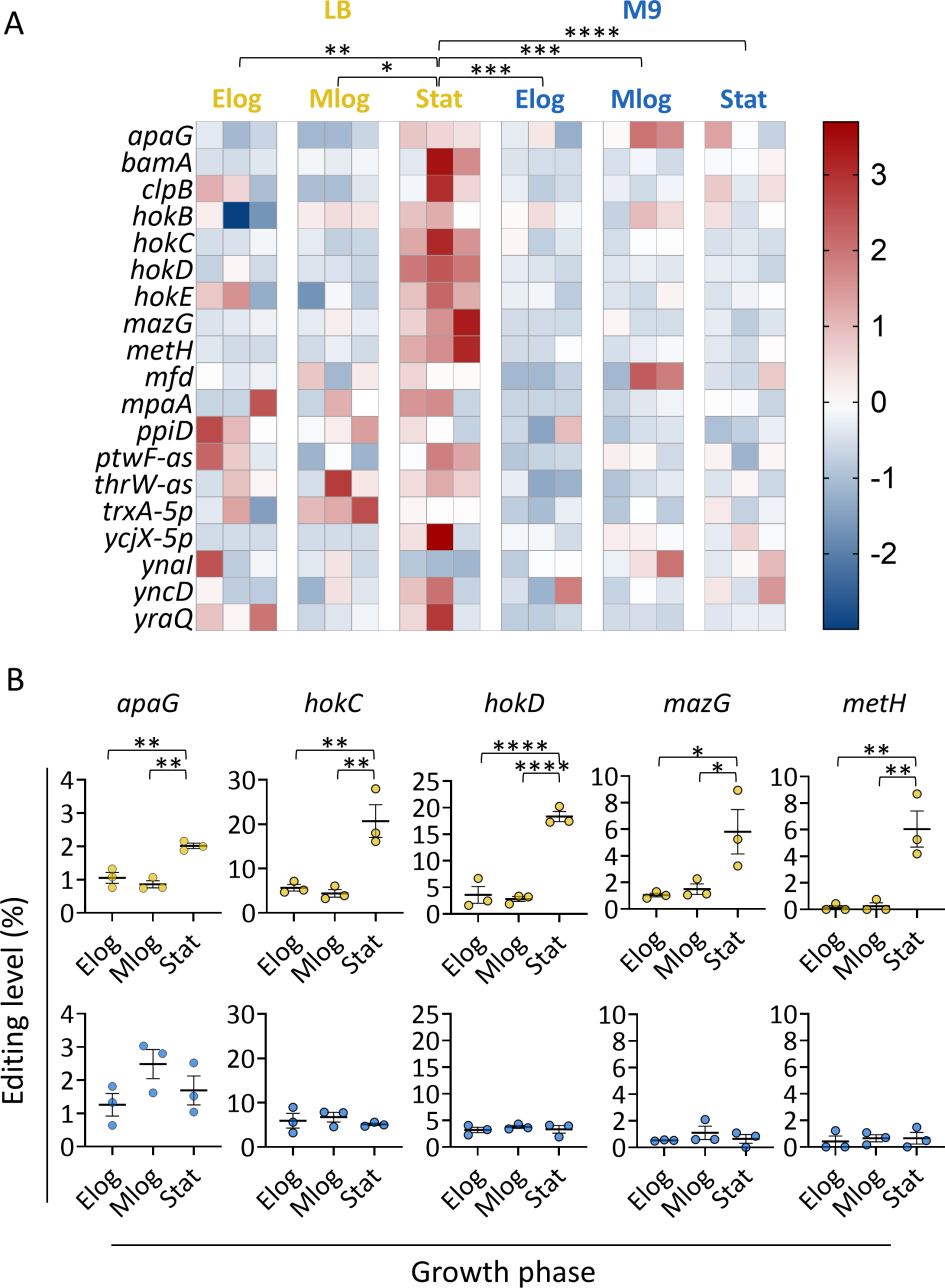
Growth media and growth phase affect A-to-I RNA editing levels. (**A**) Heatmap of normalized editing levels (z-score) of 19 edited mRNAs as measured across growth phases in LB (yellow) and M9 (blue). Statistical analysis was conducted using two-way ANOVA, focusing on the difference between the growth phases, followed by Tukey’s multiple testing correction. (**B**) Editing levels of five mRNAs showed the most significant increase in editing levels at the stationary phase when grown in LB. The top panel shows editing levels when grown in LB, and the bottom panel shows editing levels when grown in M9. Statistical analysis was conducted using one-way ANOVA with Tukey’s multiple testing correction. *P* value marks (in panels **A** and **B**) are as follows: *P* ≤ 0.05 (*), *P* ≤ 0.01 (**), *P* ≤ 0.001 (***), *P* ≤ 0.0001 (****); only significant comparisons are marked.

Next, we examined whether editing levels change as a function of growth media and growth phases in culture. We analyzed the editing levels, separating each data set according to growth phase and medium. We observed that global mRNA editing levels significantly increased in the stationary phase compared to the early- and mid-log phases when bacteria grew in LB (Fig. 2A; Table S4). In contrast, no significant change was observed when bacteria grew in M9 (Fig. 2A). Moreover, editing levels were also significantly higher in LB at the stationary phase than in all growth phases in M9 (Fig. 2A). Notably, some transcripts displayed a more significant increase in editing levels than others, which showed a relatively steady level of editing across growth phases (Fig. 2B; Fig. S5 and S6; Table S4). Finally, tRNA-Arg2 editing levels did not change significantly across growth phases in bacteria grown in LB or M9 (Fig. S7; Table S5). Thus, culture’s growth medium and phase can regulate A-to-I mRNA editing levels in *E. coli*.

### tRNA-Arg2 expression is significantly downregulated at the stationary phase

**Fig 3:**
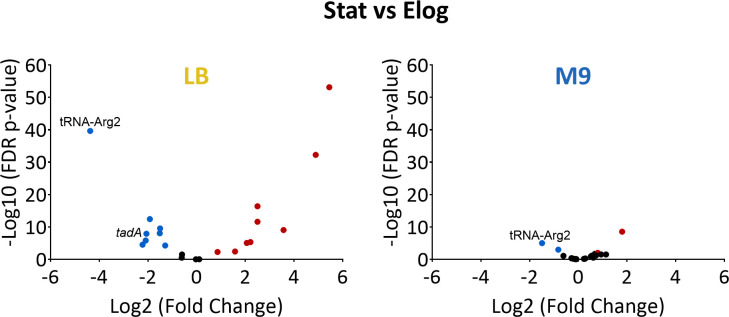
tRNA-Arg2 expression is significantly downregulated at the stationary phase. Volcano plots showing the change in gene expression of 19 edited mRNAs, tRNA-Arg2, and *tadA* between samples in the stationary phase and early logarithmic phase in either LB or M9. Significant values (false discovery rate [FDR] *P* value ≤ 0.01) are marked in blue and red for downregulated and upregulated genes, respectively. Values used for Fig. 3 are found in Table S6.

Next, we examined what molecular factors can affect observed changes in mRNA editing levels. First, we decided to test for a link between mRNA editing and the expression of edited mRNAs, tRNA-Arg2, or *tadA*. In contrast to our observation that editing levels increase at the stationary phase in LB, we did not observe a clear trend regarding edited mRNA expression in the stationary phase compared to the early logarithmic phase (Fig. 3; Table S6). Specifically, we observed that 9/19 transcripts were significantly upregulated, 6/19 significantly downregulated, and 4/19 were unchanged (Fig. 3; Table S6). A similar trend was observed when we examined the change in gene expression between the stationary and mid-log phase (Fig. S8). In contrast to LB, when bacteria grew in M9, only two edited mRNAs were significantly upregulated in the stationary phase, and one was significantly downregulated (Fig. 3; Table S6). We conclude that mRNA expression levels cannot account for the increased mRNA editing level at the stationary phase in LB.

When we examined the expression level of *tadA*, we observed that it was significantly downregulated at the stationary phase compared to the early log phase in LB (Fig. 3). This was counterintuitive to our observation that editing increases at the stationary phase in LB. However, we also observed that tRNA-Arg2 expression, the primary substrate of TadA, was the most significantly downregulated RNA at the stationary phase compared to the early- and mid-log phases when grown in LB (Fig. 3; Fig. S8; Table S6). Notably, in M9, the reduction in tRNA-Arg2 expression in the stationary phase was very moderate, albeit significant (Fig. 3; Fig. S8; Table S6). Thus, reduced tRNA-Arg2 expression might be linked to increased mRNA editing levels at the stationary phase in LB.

### Increased tRNA-Arg2 expression reduced global RNA editing levels

Because A-to-I RNA editing levels increased while tRNA-Arg2 expression decreased at the stationary phase in LB, we aimed to test if altering tRNA-Arg2 expression can affect editing levels.

**Fig 4:**
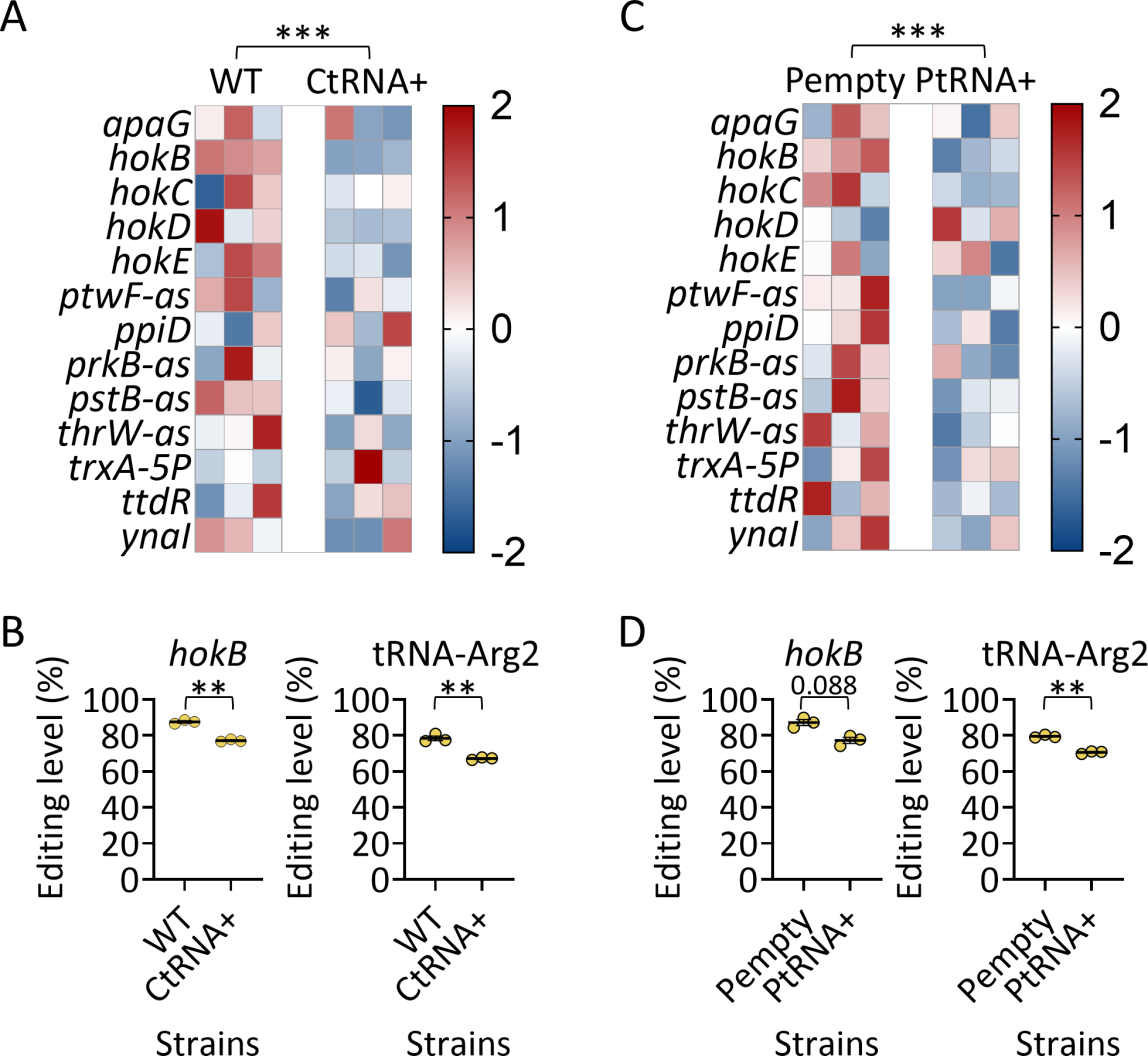
Increased tRNA-Arg2 expression reduced RNA editing levels. (**A**) Heatmap of z-score-normalized mRNA editing levels from *E. coli* expressing an additional tRNA-Arg2 from a chromosomal origin (CtRNA+) and control (WT). (**B**) RNA editing levels in *hokB* and tRNA-Arg2 in the WT and CtRNA+ strains. (**C**) Heatmap of z-score-normalized mRNA editing levels from *E. coli* overexpressing tRNA-Arg2 from a plasmid origin and control (Pempty). (**D**) RNA editing levels in *hokB* and tRNA-Arg2 in the Pempty and PtRNA+ strains. Statistical analysis in panels A and C was conducted using two-way ANOVA, focusing on the difference between the strains. Student’s or Welch’s *t*-test was used to analyze all genes shown in panels A and C, followed by Benjamini-Hochberg FDR correction (Tables S9 and S11), and relevant values are marked in panels B and D. *P* value marks are as follows: *P* ≤ 0.01 (**) and *P* ≤ 0.001 (***). In all panels, three biological replicates are shown (*n* = 3) of bacteria that were grown on LB to mid-log phase prior to RNA extraction.

First, we examined whether increased tRNA-Arg2 expression can affect RNA editing levels. Thus, we used a strain with a duplicated operon of the four tRNA-Arg2 genes *argVYZQ* (on the chromosome at the *araBAD* locus; termed CtRNA+) and performed RNA-seq to examine changes in editing levels compared to the WT strain. Importantly, we observed a 2.4-fold increase in tRNA-Arg2 expression in CtRNA+ compared to WT (Table S7). Moreover, we identified two additional editing events, not identified by our initial analysis, shown in Fig. 1: one previously reported by us (in *prkB-as*), and another never reported (in *pstB-as*). Also, some of the identified editing events described in Fig. 1 were not identified, again supporting that a subset of RNA editing events can vary between strains, samples, and experiments. Finally, we observed a decrease in global A-to-I RNA editing levels in the CtRNA+ compared to the WT strain (Fig. 4A; Tables S8 and S9). Moreover, editing levels in *hokB* and tRNA-Arg2 decreased significantly in the CtRNA+ compared to the WT strain (Fig. 4B; Tables S8 and S9).

To further investigate if the increased tRNA-Arg2 expression can decrease RNA editing levels, we overexpressed tRNA-Arg2 from a plasmid and compared the RNA editing levels to the same strain having an empty plasmid as a control (termed PtRNA+ and Pempty, respectively). Here, we observed a 13.4-fold increase in tRNA-Arg2 expression in PtRNA+ compared to the Pempty strain (Table S7). Subsequently, we observed decreased global A-to-I RNA editing levels in the PtRNA+ compared to the Pempty strain (Fig. 4C; Tables S10 and S11). Here, editing levels in *hokB* and tRNA-Arg2 decreased in the PtRNA+ compared to the Pempty strain, although marginally significant in the case of *hokB* (Fig. 4D; Tables S10 and S11).

We conclude that increased tRNA-Arg2 levels can affect RNA editing levels globally (across all RNAs) and in specific RNAs more than in others (in *hokB* and tRNA-Arg2).

### Decreased tRNA-Arg2 expression affects *hokB* and tRNA-Arg2 editing levels

Next, we examined whether decreased tRNA-Arg2 expression can affect RNA editing levels. Thus, we used a strain with only one copy of tRNA-Arg2 in its genome (*argV*; termed CtRNA−) by deleting three tRNA-Arg2 genes (*argZ*, *argY*, and *argQ*) and performed RNA-seq to examine changes in editing levels compared to the WT strain. Importantly, we observed a 16.1-fold decrease in tRNA-Arg2 expression in CtRNA− compared to the WT (Table S7). Here, we identified three additional editing events not detected by previous analysis in this manuscript: two previously reported by us (*speD* and *treC*), and another that was never reported (*yccM-as*). Here as well, some of the identified editing events described previously or within the current work were not identified, further supporting that a subset of RNA editing events can vary between strains, samples, and experiments.

**Fig 5:**
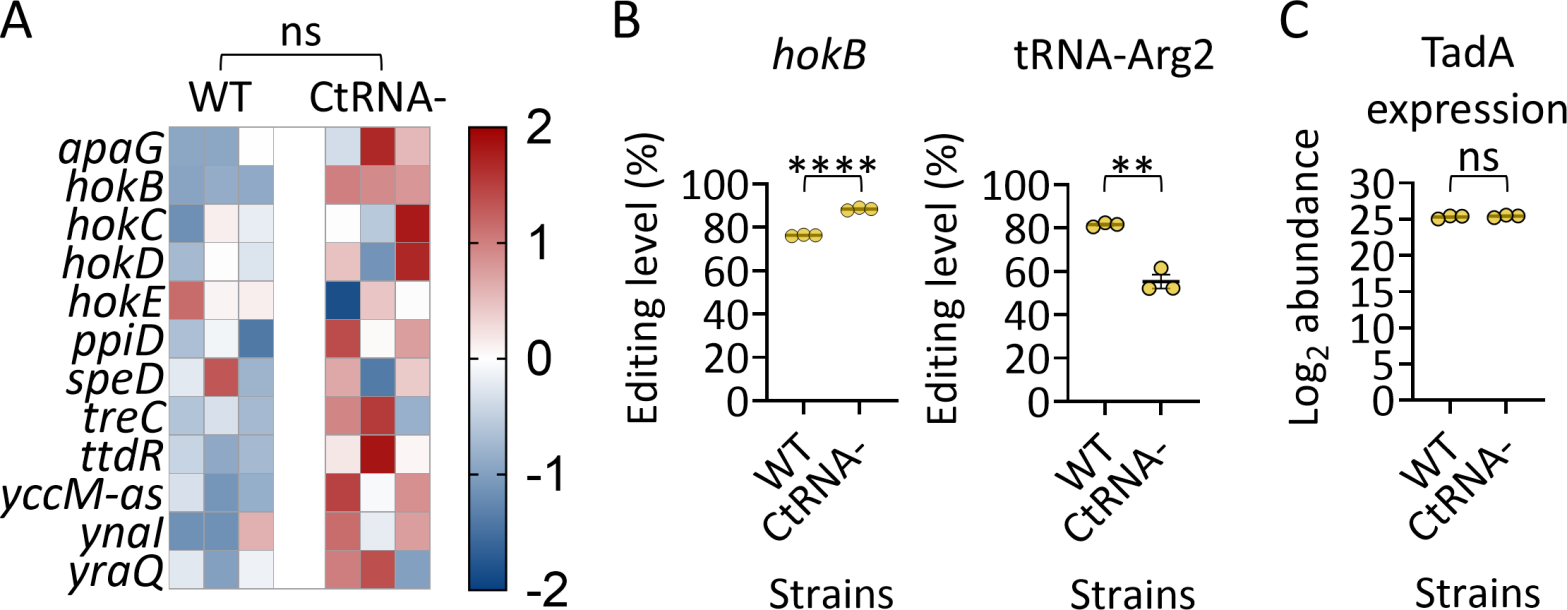
Decreased tRNA-Arg2 expression affects *hokB* and tRNA-Arg2 editing levels. (**A**) Heatmap of z-score-normalized mRNA editing levels from *E. coli* strain with only one copy of tRNA-Arg2 in its genome (CtRNA−) and control (WT). (**B**) RNA editing levels in *hokB* and tRNA-Arg2 in the WT and CtRNA− strains. (**C**) TadA protein expression levels, as measured by protein mass spectrometry, are shown as TadA total log_2_ protein abundance. Statistical analysis in panel A was conducted using two-way ANOVA, focusing on the difference between the strains. Student’s or Welch’s *t*-test was used to analyze all genes shown in panels A and B, followed by Benjamini-Hochberg FDR correction (Table S13), and relevant values are marked in panel B. Student’s or *t*-test was used to analyze TadA expression in panel **C**. *P* value marks are as follows: *P* ≤ 0.01 (**) and *P* ≤ 0.0001 (****). In all panels, bacteria were grown in LB to mid-log phase prior to RNA extraction.

We detected only a minor increase in global editing levels in the CtRNA− compared to the WT strain (Fig. 5A; Tables S12 and S13). However, when we tested for differences in editing levels between individual RNAs, we observed that editing of *hokB* increased and editing of tRNA-Arg2 decreased significantly in the CtRNA− strain compared to the WT strain (Fig. 5B; Tables S12 and S13).

The gene encoding TadA contains 14 tRNA-Arg2-dependent codons, raising the possibility that TadA expression itself could be linked to tRNA-Arg2 expression and editing, thereby accounting for the moderate increase in global editing levels and the decrease observed in tRNA-Arg2 editing. To examine this possibility, we performed protein mass spectrometry and detected no significant difference in TadA protein levels between the WT and CtRNA− strains (Fig. 5C; Table S15). We conclude that decreased tRNA-Arg2 expression can affect RNA editing levels in specific RNAs, and that TadA expression is not affected by reduced tRNA-Arg2 expression or editing.

### tRNA-Arg2 editing is important for growth in nutrient-rich media

**Fig 6:**
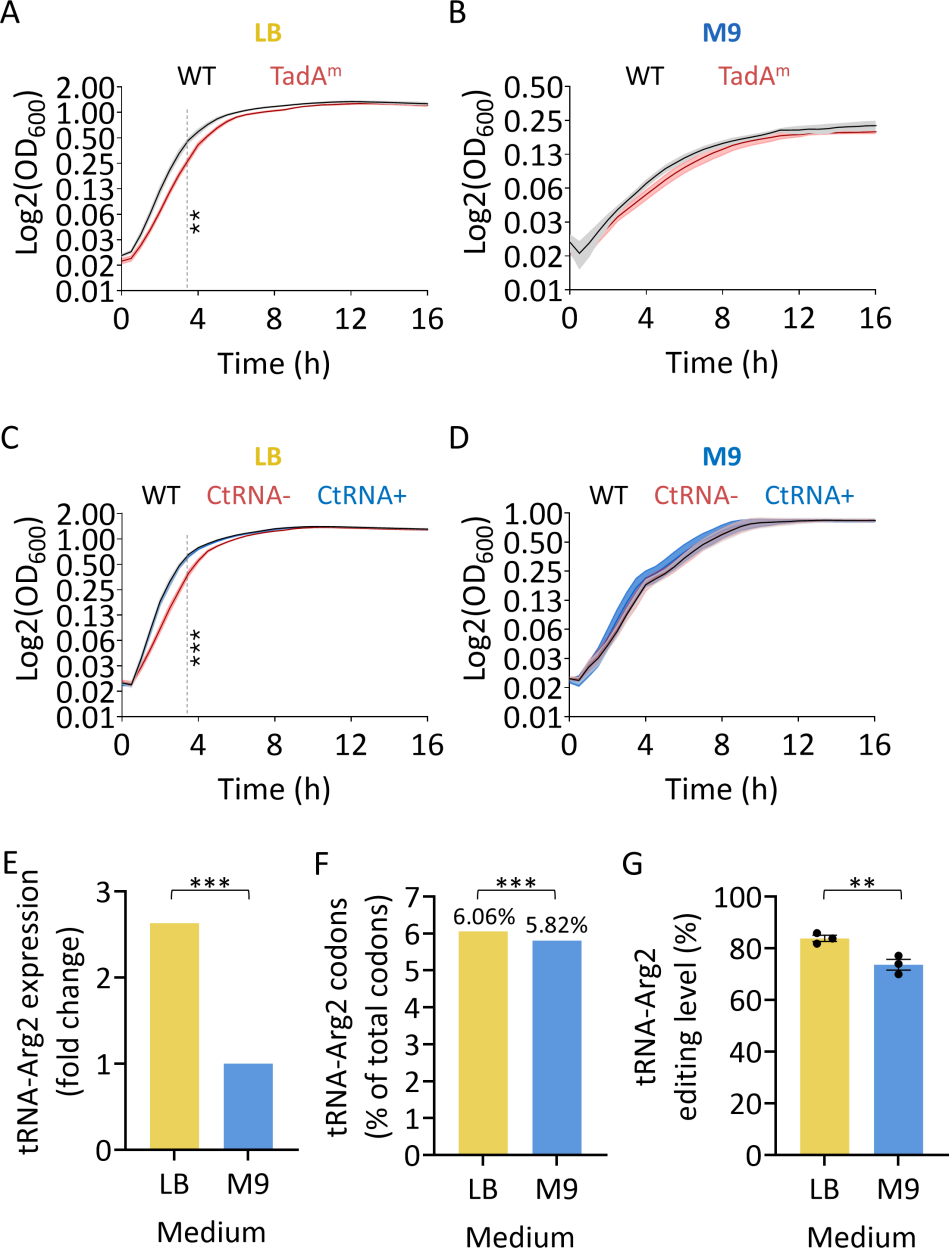
tRNA-Arg2 editing is important for growth in nutrient-rich media. (**A**) Growth of WT and TadA^m^ *E. coli* strains in LB at 34°C. (**B**) Growth of WT and TadA^m^ strains in M9 at 34°C. (**C**) Growth of WT, CtRNA−, and CtRNA+ strains in LB at 37°C. (**D**) Growth of WT, CtRNA−, and CtRNA+ strains in M9 at 37°C. (**E**) tRNA-Arg2 expression was calculated from differential gene expression analysis of bacteria grown in LB versus M9 at mid-log phase. (**F**) Frequency of tRNA-Arg2-dependent codons (CGU, CGA, CGC) in genes upregulated in LB versus M9 and vice versa. (**G**) tRNA-Arg2 editing levels in bacteria grown in LB and M9 at the mid-log phase. The mean (solid line) and standard error (shaded area) of three biological replicates performed on different days (*N* = 3), each with 21 or 28 technical replicates, are shown. The dashed gray line at 3.5 h marks the end of the log phase in the WT strain and was used for statistical comparisons. Statistical analysis was conducted using Student’s *t*-test (**A** and **B**); one-way ANOVA (**C** and **D**), followed by Tukey’s multiple-comparisons test, which revealed a significant difference between the CtRNA− strain and the WT (***) and CtRNA+ (**) strains; false discovery rate-adjusted *P* values from differential gene expression analysis (**E**); two-sided Fisher’s exact test (**F**); and, one-way ANOVA, followed by Šídák’s multiple-comparisons test (**G**), as part of the analysis shown in Fig. S9. Only significant values are shown in all panels: *P* value ≤ 0.01 (**); ≤ 0.001 (***).

Next, we sought to test the functional consequences of a TadA mutant with reduced tRNA and mRNA editing activity in *E. coli* (Fig. 1). Therefore, we compared the growth of the WT and TadA^m^ strains in LB and M9. Under nutrient-rich conditions (LB), the TadA^m^ strain showed a moderate but significant growth defect (Fig. 6A), whereas no significant difference in growth was observed between the WT and TadA^m^ strains in nutrient-limited M9 medium (Fig. 6B). Similarly, the CtRNA− strain displayed a comparable growth defect relative to the WT and CtRNA+ strains in LB, but not in M9 (Fig. 6C and D).

Consistent with these phenotypes, differential expression analysis revealed that tRNA-Arg2 levels are significantly higher in LB than in M9 (Fig. 6E; Table S15). In addition, codon usage analysis showed that genes upregulated in LB contain significantly more tRNA-Arg2-dependent codons (CGU, CGA, CGC) than genes upregulated in M9 (Fig. 6F; Table S15). Finally, tRNA-Arg2 editing levels were significantly higher in LB than in M9 across all growth phases (Fig. 6G; Fig. S9). Together, these results indicate that reduced tRNA-Arg2 editing phenocopies reduced tRNA-Arg2 expression and support the conclusion that proper tRNA-Arg2 editing is important for optimal growth under nutrient-rich conditions.

## DISCUSSION

Our study reveals that A-to-I mRNA editing is a dynamic and possibly regulated process in *E. coli*. Moreover, our work increases the repertoire of known A-to-I mRNA editing events to 27 sites and supports the notion that TadA activity is important for optimal growth in *E. coli*.

What can explain the increase in editing levels observed at the stationary phase? Conditions are dramatically altered at the stationary phase compared to the logarithmic phase. Among others, bacterial density increases, nutrients become scarce, and waste products accumulate. To adapt to these environmental changes, bacteria alter their metabolism and morphology (32–34). Accordingly, our work supports the idea that bacteria also increase their mRNA editing levels at the stationary phase. Thus, some of the stress conditions experienced during the stationary phase might induce changes in editing levels. Indeed, stress conditions (e.g., oxidative stress) were shown to modulate A-to-I mRNA editing in *S. pyogenes* (24). However, in contrast to *E. coli*, editing levels were not significantly altered between growth phases in *S. pyogenes* (24). Thus, it is plausible that A-to-I editing could be regulated by different factors in different species (35).

In a recent report, we demonstrated that RNA editing in *hokB*, which encodes a toxin in the HokB-*sokB* toxin-antitoxin system, can recode the sequence of HokB, modulate its function, and regulate disulfide bond formation (36). We focused on *hokB* because it is the most highly edited mRNA reported in bacteria to date (Fig. 1G). However, the functional relevance of editing in other transcripts—particularly those with low editing levels—remains unclear. An observed editing level of 5% may reflect differences between individual cells rather than variation among RNA molecules within a single bacterium. Thus, 5% of the population will harbor only the edited version, while the rest will encode only the non-edited version of a particular mRNA. Such cell-to-cell heterogeneity could contribute to phenotypic diversity among genetically identical bacteria, potentially enhancing their resilience to environmental fluctuations. Nevertheless, further research is required to test this hypothesis, ideally through single-cell RNA sequencing combined with functional assays tailored to specific editing events.

Previously, we showed that overexpressing TadA elevated mRNA editing levels (23). Thus, increasing TadA’s availability can affect editing levels in bacteria. Another way to increase the available TadA units is to change the newly synthesized, pre-edited tRNA-Arg2 expression. In other words, we and others have hypothesized that tRNA-Arg2 may compete with mRNAs for TadA availability, limiting its ability to edit mRNAs (24, 37). Indeed, when we overexpressed tRNA-Arg2 from a chromosomal or plasmid source, global editing levels decreased. Interestingly, overexpressing tRNA-Arg2 from a plasmid increased tRNA-Arg2 levels by an order of magnitude more than chromosomal upregulation, yet the reduction in mRNA editing levels was similar. This suggests that the expression source or genomic context may influence the accessibility of tRNA-Arg2 to TadA and thus its impact on mRNA editing levels. Alternatively, chromosomal upregulation of tRNA-Arg2 already occupies TadA to an extent that saturates the enzyme and leaves very little activity for mRNA editing.

In contrast to increasing tRNA-Arg2 expression, reducing tRNA-Arg2 expression did not result in a marked increase in mRNA editing levels. One possibility is that the decrease in tRNA-Arg2 expression was insufficient to elicit a stronger effect on mRNA editing levels; that is, despite the reduction in total tRNA-Arg2, there may still be enough molecules to effectively compete with mRNA substrates and limit their editing. Moreover, editing of the remaining tRNA-Arg2 copy encoded by *argV* decreased in the CtRNA− strain, lacking three out of four tRNA-Arg2 genes (*argQ*, *argY*, and *argZ*). It is tempting to speculate that the presence of four tRNA-Arg2 copies arranged in tandem within the same operon may facilitate efficient recruitment of TadA, thereby promoting higher tRNA editing activity under wild-type conditions. Finally, substrate specificity might also limit the response of mRNA editing to changes in tRNA-Arg2 levels: even if TadA abundance is already sufficient, its affinity for mRNA substrates may be lower than for tRNA-Arg2, constraining any further increase in mRNA editing.

Another interesting observation is that tRNA-Arg2 editing levels were almost completely abolished in the TadA^m^ when grown in M9. Despite this, we did not observe a significant difference between the WT and TadA^m^ strains in our growth assays in M9 medium. This result is surprising, as TadA editing of tRNA-Arg2 was considered essential in bacteria (22). It could be that having unedited tRNA-Arg2 is not essential under nutrient-limiting conditions. Alternatively, it could be that TadA has yet-to-be-discovered cryptic activity that underlies its essentiality.

In contrast to M9, when bacteria were grown on LB, the WT grew faster than the TadA^m^ strain. It is tempting to speculate that the TadA mutation has a more severe effect on tRNA-Arg2, slowing translation at tRNA-Arg2-dependent codons, similar to what has been observed when wobble-position modifications are perturbed in other tRNAs (38). That would be the case during log phase growth in LB, where ribosomes move at up to 20 amino acids per second, whereas it is maybe less important in M9, where ribosomes move more slowly (10–13 amino acids per second) (39). Consistent with a tRNA-Arg2-dependent effect, loss of three tRNA-Arg2 gene copies phenocopies the TadA^m^ growth defect, supporting the idea that reduced tRNA editing functionally resembles decreased tRNA-Arg2 availability. Moreover, tRNA-Arg2 expression is elevated in LB relative to M9, and genes upregulated in LB relative to M9 contain more tRNA-Arg2-dependent codons. Taken together, these observations suggest that tRNA-Arg2 editing supports efficient translation of genes required for rapid growth under nutrient-rich conditions. Importantly, although the difference in growth was moderate (but significant), it should be considered that our experimental setup allowed about 6–7 replication cycles. Thus, on a longer time scale, when we consider competition between bacterial strains, it is clear that this moderate difference could become substantial. Lastly, because the TadA^m^ strain harbors additional mutations (see Materials and Methods), we cannot exclude the possibility that they contribute to the observed growth phenotype in LB. However, the cumulative results described above support the view that tRNA-Arg2 editing is a major driver of the growth phenotype in LB.

Finally, many questions remain open regarding A-to-I mRNA editing regulation in bacteria. For example, future research should examine whether TadA is the only A-to-I editing enzyme in bacteria; if other proteins interact with TadA to regulate its activity; and the existence of other proteins that interact with or process TadA-dependent edited RNAs.

Overall, our study expands our understanding of bacterial RNA editing by demonstrating its regulation through intrinsic (molecular) and extrinsic (environmental) factors. Finally, our work lays the foundation for studying the regulation and functional role of mRNA editing in other bacterial species.

## MATERIALS AND METHODS

### Bacterial strains, DNA and RNA extractions, and cDNA synthesis

In the current work, we used the following strains: *E. coli* MG1655-EcM2.1 (WT and TadA^m^), MAS1081 (WT), MAS1100 (CtRNA−), TSS248 (CtRNA+), TSS50 (Pempty), TSS253 (PtRNA+) (23, 40). The TadA^m^ strain was created using Multiplex Automated Genome Engineering, which carries a loss-of-function mutation in *mutS* (23, 41). Consequently, the TadA^m^ strain harbors seven additional mutations in addition to the desired mutation in *tadA* (Table S16). Despite our multiple attempts, we were not able to recreate the TadA^m^ allele using alternative methods, possibly suggesting that these additional mutations complement or protect against the full-scale deleterious effect of the mutation in TadA. Deletion of chromosomal *argYZQ* [Δ(2815672-2816445)] and insertion of the *serV-argVYZQ* operon at the *araBAD* locus were done by recombineering (42). The chromosomal changes were first constructed in an *E. coli* MG1655 derivative (MAS1080) harboring the λ RED defective prophage [λcI_857_ Δ(*cro‐bioA*)], as described (42), and then moved to the MAS1081 strain background by P1 transduction to generate strains MAS1100 and TSS248. Strains TSS50 and TSS253 are MAS1081 containing the pIDA1 vector (Pempty), or the pIDA1 vector expressing *argV* from the Ptac promoter (PtRNA+) (43).

Bacteria were grown on LB medium (10 g tryptone, 10 g sodium chloride, and 5 g yeast extract per liter) or M9 medium (200 mL M9 5× salts solution, 2 mL of 1 M magnesium sulfate [MgSO_4_], 100 uL 1 M calcium chloride [CaCl_2_], complete with DDW to 1 L, and supplemented with glucose to a final concentration of 0.2%). *E. coli* MG1655-EcM2.1 (WT and TadA^m^) strains were grown at 34°C, and the rest at 37°C. For the PtRNA+ and Pempty strains, isopropyl β-D-1-thiogalactopyranoside was added at the beginning of the experiment at a final concentration of 1 mM. At early log (OD_600_ of 0.2), mid-log (OD_600_ of 0.8), or stationary phase (24 h of growth), 1 mL was taken for DNA and RNA extractions. DNA was extracted using a GeneJET Genomic DNA Purification Kit (Thermo Scientific #K0721) or the iNtRON biotechnology G-spin Genomic DNA Purification Kit (Life gene # 17121), while RNA was extracted using the GeneJET RNA Purification Kit (Thermo Scientific # K0731). RNA samples were treated with 4 units of DNase I (NEB # M0303L) for 20 min at 37°C. Finally, following incubation of 15 min at 65°C, cDNA synthesis was performed using GoScript Reverse Transcription Mix (Promega # A2801). To synthesize cDNA, 500 ng of total RNA from *E. coli* strains were primed with random hexamers and reverse-transcribed with the GoScript Reverse Transcription Mix kit (A2801 Promega) following the manufacturer’s protocol.

### RNA-seq, DNA-seq, and RNA editing analysis

Samples were grown in LB medium or M9, each started from a single and different colony. RNA was extracted at early log (OD_600_ of 0.1–0.2), mid-log (OD_600_ of 0.7–1.0), or stationary phase (24 h of growth), as described above. Ribosomal RNA was depleted using NEBNext rRNA Depletion Kit (Bacteria) (New England Biolabs, #E7850). RNA-seq libraries were constructed using NEBNext Ultra II Directional RNA Library Prep Kit for Illumina (New England Biolabs, #E7760). DNA-seq libraries were prepared using NEBNext Ultra II FS DNA Library Prep Kit for Illumina (New England Biolabs, #E7805L). Finally, RNA-seq libraries were sequenced on the NovaSeq X platform (Illumina).

We used CLC Genomics Workbench for all analysis steps (described below).

RNA-seq reads were first trimmed according to length and quality scores to ensure high quality of the reads by using the following parameters: Trim using quality scores = Yes; Quality limit = 0.01; Trim ambiguous nucleotides = Yes; Maximum number of ambiguities = 1; Automatic read-through adapter trimming = Yes; Minimum length = 50; Maximum length = 150; Remove 5′ terminal nucleotides = No; Remove 3′ terminal nucleotides = No; Remove on first read = Yes; Remove on second read (for paired reads) = Yes; Trim to a fixed length = No; Trim end = Trim from 3′-end; Discard short reads = Yes; Discard long reads = No; Save discarded sequences = No; Save broken pairs = No.

Paired RNA reads were merged into a single longer read, maintaining the original orientation of the R1 reads (reverse to the original transcript direction). The parameters were as follows: Mismatch cost = 2; Minimum score = 8; Gap cost = 3; Maximum unaligned end mismatches = 0.

Next, RNA-seq reads were mapped to the NC_000913.3 *E. coli* reference genome with the following parameters: Masking mode = No masking; Match score = 1; Mismatch cost = 2; Cost of insertions and deletions = Linear gap cost; Insertion cost = 3; Deletion cost = 3; Length fraction = 0.95; Similarity fraction = 0.95; Global alignment = No; Non-specific match handling = Ignore.

Initial variant calling was performed using the following parameters: Ignore positions with coverage above = 100,000; Restrict calling to target regions = Not set; Ignore broken pairs = Yes; Ignore non-specific matches = Reads; Minimum coverage = 4; Minimum count = 2; Minimum variant frequency (%) = 0.1; Base quality filter = Yes; Neighborhood radius = 5; Minimum central quality = 30; Minimum neighborhood quality = 30; Read direction filter = No; Relative read direction filter = No; Read position filter = No.

When bacteria grew in LB vs M9, variants were filtered against the DNA samples. Only variants not found in the DNA-seq data set were kept.

After the initial variant calling was performed, additional filtering was applied. All filtered variants were required to match all the following criteria: Criteria = “Type contains SNV”; Criteria = “Reference allele contains No”; Criteria = “Frequency (editing level) >= 1%”; Criteria = “# unique start positions >= 3”; Criteria = “# unique end positions >= 3.”

For sites encoded from the positive strand: Criteria = “Reverse read count >= 3”; Criteria = “Reverse read coverage >= 10”; Criteria = “Reference contains A and the variant contains G.”

For sites encoded from the negative strand: Criteria = “Forward read count >= 3”; Criteria = “Forward read coverage >= 10”; Criteria = “Reference contains T and the variant contains C.”

Next, we filtered variants shared between at least two (out of three) biological replicates of each growth phase.

All the main steps in the RNA editing analysis are found in Fig. S1.

Finally, we extracted the status of the identified shared variants from the RNA mapping step to ensure we did not miss variants because of our filters and to ensure that when a variant is absent in our initial analysis, it is not because its transcript was not sequenced.

### Sequence motif and secondary structure prediction around editing events

We used WebLogo to identify the sequence motif around editing events (44). We used RNAfold from the ViennaRNA Package 2.0 (31) to calculate the minimum free energy around editing events. As previously shown on editing events in *S. pyogenes* (24), we calculated the structure of the minimum free energy of 17 nucleotides around edited sites (which is the length of *tRNA^Arg2^* anticodon arm) and of 37 nucleotides around edited sites. We positioned the edited adenosine at position “0” in our sliding window analysis, similar to its location in the anticodon arm of *tRNA^Arg2^*.

### Differential gene expression analysis

First, we extracted the sequences of all annotated genes in the *E. coli* genome. In addition, we added four transcripts that represent *trxA-5p*, *ycjX-5p*, *thrW-as,* and *ptwF-as*, as these were not annotated in the original genome (Fig. S10). We refer to this as the transcriptome of NC_000913.3. Next, to obtain normalized expression measurements (as transcripts per million—TPM), we mapped the RNA-seq reads (following quality control and trimming of adapter read-through) to the transcriptome with the following parameters in CLC Genomics Workbench: Use spike-in controls = no; Mismatch cost = 2; Insertion cost = 3; Deletion cost = 3; Length fraction = 0.95; Similarity fraction = 0.95; Global alignment = No; Strand specific = Reverse; Library type = Bulk; Maximum number of hits for a read = 10; Count paired reads as two = No; Ignore broken pairs = Yes; Expression value = TPM. Finally, we analyzed differential gene expression with default parameters (also using CLC Genomics Workbench).

### Growth assays

*E. coli* cultures were grown at 34°C for 24 h in LB medium, back-diluted in a 1:100 ratio to a 50 mL conical tube containing either 10 mL LB or M9 medium (the latter supplemented with 0.2% glucose, final concentration), vortexed, and dispensed (150 µL) to 96-well plates (Corning Costar). Wells were measured every 30 min for optical density at OD_600_ for 24 h (Synergy H1, Biotek). The 96-well plate was divided as follows: 12 wells were blank control (line A of the plate), and 42 wells (half of the remaining plate) were divided between the WT and TadA^m^ strains. For each strain, growth curves were obtained from 14 to 21 technical replicates (wells in the plate). We conducted three independent experiments (biological repeats) at 34°C, on different days, with starters from different colonies.

### Codon usage analysis

Differential gene expression analysis was first performed as described above using RNA-seq data from mid-log-phase bacteria grown in LB and M9. Next, we extracted the coding sequences of all genes that were upregulated in either LB or M9. Finally, we analyzed all open reading frames with the Kazusa codon usage web server to calculate codon usage in upregulated genes in LB vs M9 and vice versa (45).

### PCR of DNA and cDNA samples of *E. coli*

RNA and DNA were extracted at the stationary phase (OD600 > 3) from *E. coli* as described above. PCR was conducted using primers found in Table S17. The samples were cleaned using the Zymo DNA Clean & Concentrator kit (#D4004), then sent for Sanger sequencing.

### Proteolysis

Bacterial cells were lysed in 8.5 M urea, 400 mM ammonium bicarbonate, and 10 mM DTT, sonicated twice (90%, 10–10, 5 min), and centrifuged (10,000 × *g*, 10 min). Protein amount was estimated using Bradford readings. The samples were reduced (60°C for 30 min), modified with 35.2 mM iodoacetamide in 100 mM ammonium bicarbonate (room temperature for 30 min in the dark), and digested in 1.5 M urea, 66 mM ammonium bicarbonate with modified trypsin (Promega), overnight at 37°C in a 1:50 (M/M) enzyme-to-substrate ratio. An additional second digestion with trypsin was done for 4 h at 37°C in a 1:100 (M/M) enzyme-to-substrate ratio. The tryptic peptides were desalted using Oasis HLB 96-well µElution Plate (Waters), dried, and re-suspended in 0.1% formic acid in 2% acetonitrile.

### Mass spectrometry analysis

The resulting peptides were analyzed by LC-MS/MS using an Exploris 480 mass spectrometer (Thermo) fitted with a capillary UHPLC (Vanquish Neo, Thermo Scientific).

The peptides were loaded in solvent A (0.1% formic acid in water) on a C18 reversed phase analytical column (Ionoptics, AUR3-25075C18-XT, 25 cm × 75 μm ID, 1.7 μm).

The peptide mixture was resolved with a 6% to 34% linear gradient of solvent B (80% acetonitrile with 0.1% formic acid in water) for 120 min, followed by a gradient of 0.1 min increase of 34% to 99% and 14 min at 99% solvent B at flow rates of 0.15 μL/min.

Mass spectrometry was performed in a positive mode using repetitively full MS scan (*m*/*z* 380–985, resolution 120,000), followed by DIA scans (10 Da isolation windows with 1 *m*/*z* overlap, and resolution 30,000).

### Data analysis 

The mass spectrometry data were analyzed using the DIA-NN software version 2.2  (46–48).

The tryptic searching against *E. coli* strain K12_UP000000625_83333 from February 2026 (4,403 entries), with minimal peptide length set to 7, maximum number of missed cleavages set to 1, cysteine carbamidomethylation enabled as a fixed modification, and protein N-term acetylation and oxidation on methionine enabled as a variable modification.

Peptide- and protein-level false discovery rates (FDRs) were filtered to 1%.  

### Statistical analysis

We used PRISM 10 or Excel to conduct the statistical analyses described above.

## Data Availability

The DNA- and RNA-seq data were deposited to the NCBI SRA (https://www.ncbi.nlm.nih.gov/sra) under accession PRJNA1269726. The mass spectrometry data were deposited to PRIDE (https://www.ebi.ac.uk/pride/) through ProteomeXchange in project accession PXD075028.

## SUPPLEMENTAL MATERIAL

Supplemental Material mbio.00551-26-s0001.docx Supplemental figures and table legends.

Supplemental Tables mbio.00551-26-s0002.xlsx Tables S1-S17.

ASM does not own the copyrights to Supplemental Material that may be linked to, or accessed through, an article. The authors have granted ASM a non-exclusive, world-wide license to publish the Supplemental Material files. Please contact the corresponding author directly for reuse.
