# Supplementary material for: Landscape and dynamics of TadA-dependent RNA editing in Escherichia coli reveal a role in nutrient-rich growth
Source: mBio. 2026 Apr 3;17(5):e00551-26. doi: 10.1128/mbio.00551-26 (PMC13170351; doi:10.1128/mbio.00551-26)
Supplement: Supplemental Material — Supplemental figures and table legends. [file mbio.00551-26-s0001.docx]

**Supplementary Figures**

**
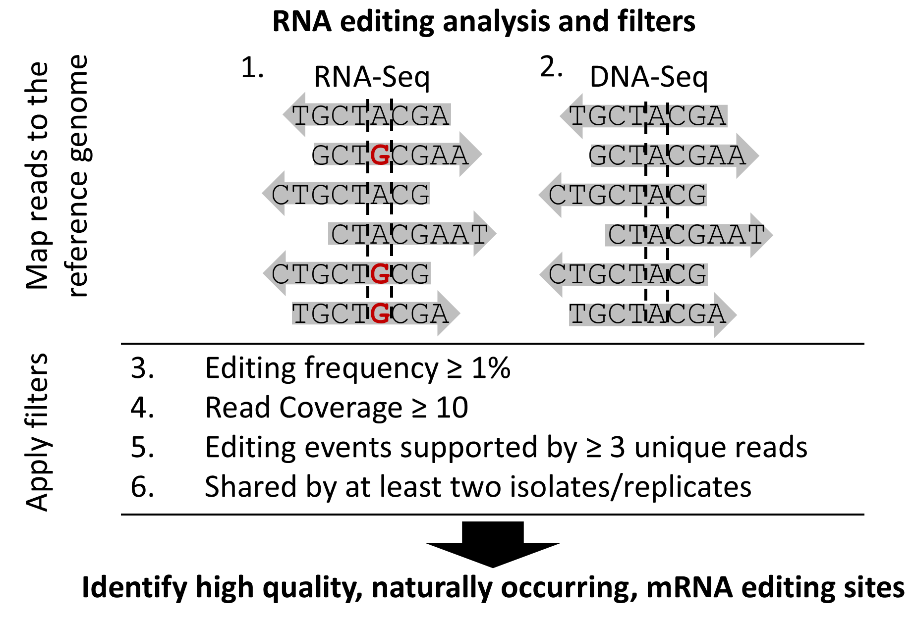
**

**Supplementary Figure 1. RNA editing analysis and filters used in this study.**

**
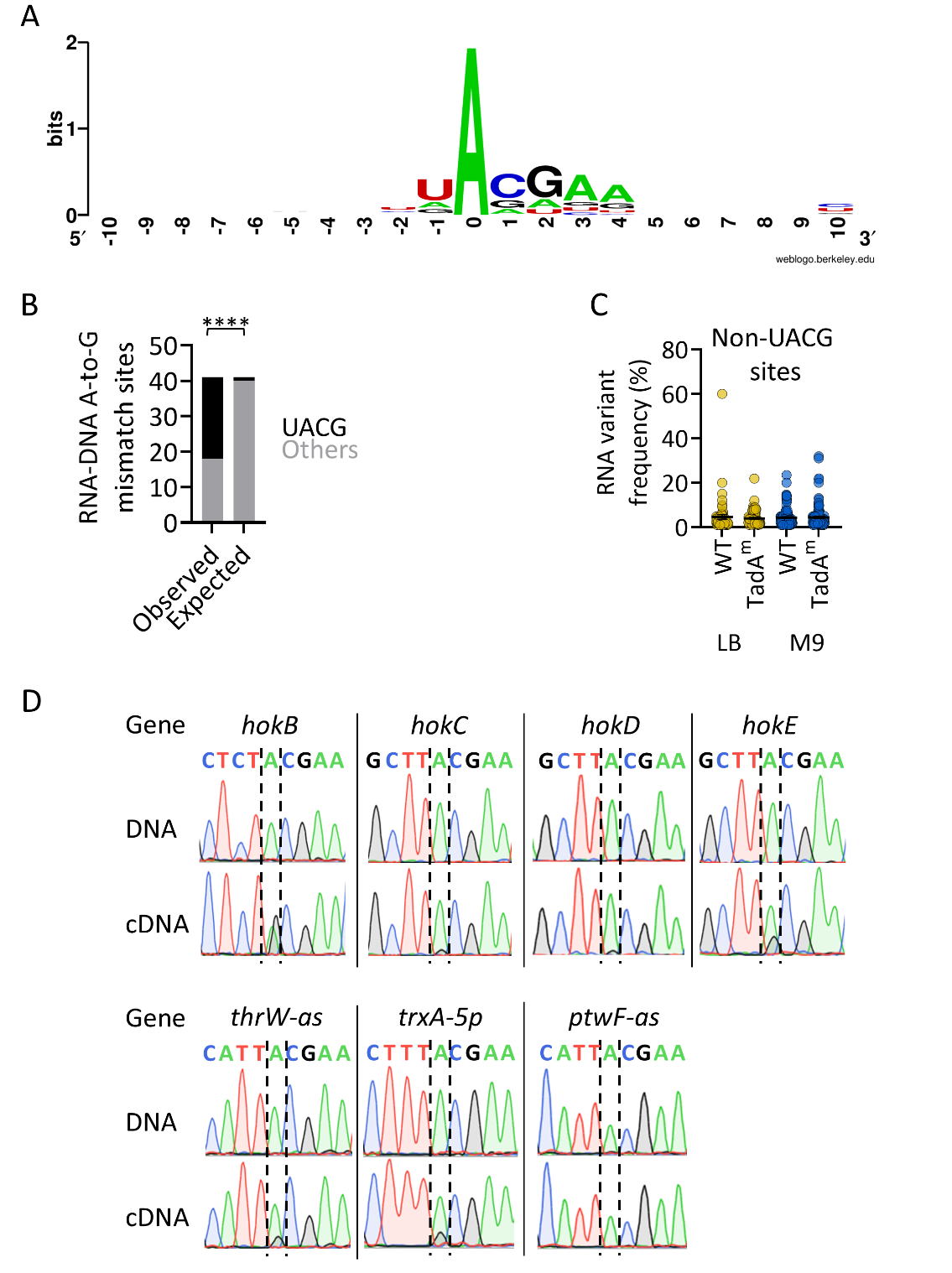
**

**Supplementary Figure 2. A-to-I mRNA editing occurs in a UACG motif and can be validated by Sanger sequencing. A.** WebLogo analysis of the 41 A-to-G RNA-DNA mismatches detected in this study *E. coli* grown in LB or M9. Position “0” is the edited site. **B**. RNA-DNA A-to-G mismatches are significantly enriched in UACG motif (Fisher’s exact test; p<0.0001). **C**. RNA-DNA A-to-G mismatches in non-UACG sites are not significantly reduced in the TadA^m^ compared to the WT strain. **D**. Sanger sequencing validates the occurrence of editing in corresponding DNA and RNA samples in 6/7 sites, including the previously reported editing event in *hokB* (also identified in the current work) that was used as a positive control. The edited site is marked with dashed lines, and the edited signal is in black at the RNA samples. Importantly, in *thrW-as* and *trxA*, we noticed variation in the edited peak between multiple attempts to validate the editing event (which could be smaller or not visible). Thus, validating editing events with Sanger sequencing can encounter biological or technical challenges.

**
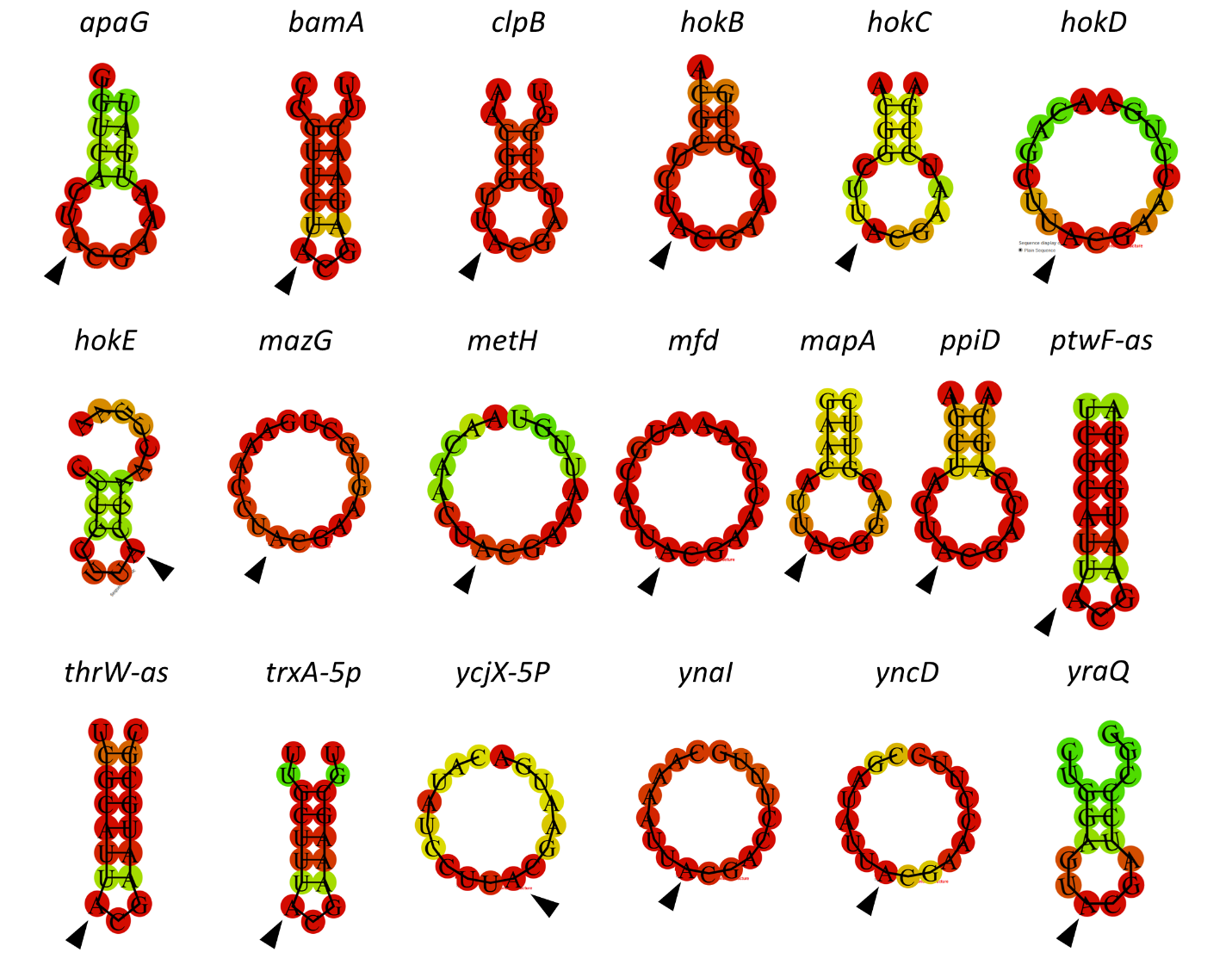
**

**Supplementary Figure 3.** Minimum free energy (MFE) secondary structure predicted by RNAfold around the A-to-I editing site (marked by a black arrow head) for the 17 nucleotides around the edited site in all 19 edited mRNAs as originally output from RNAfold.

**
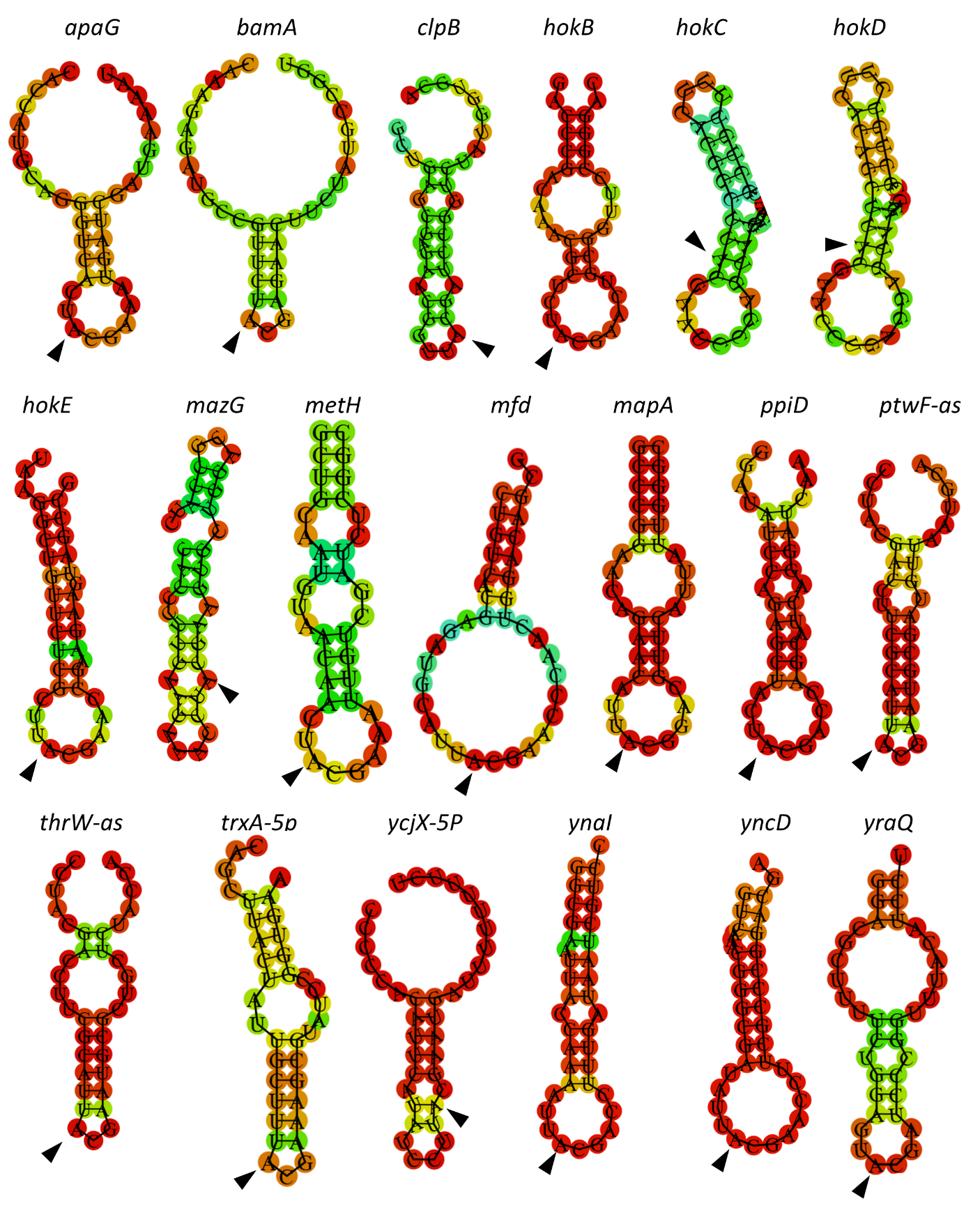
**

**Supplementary Figure 4.** Minimum free energy (MFE) secondary structure predicted by RNAfold(1) around the A-to-I editing site (marked by a black arrow head) for the 37 nucleotides around the edited site in all 19 edited mRNAs as originally output from RNAfold.

**
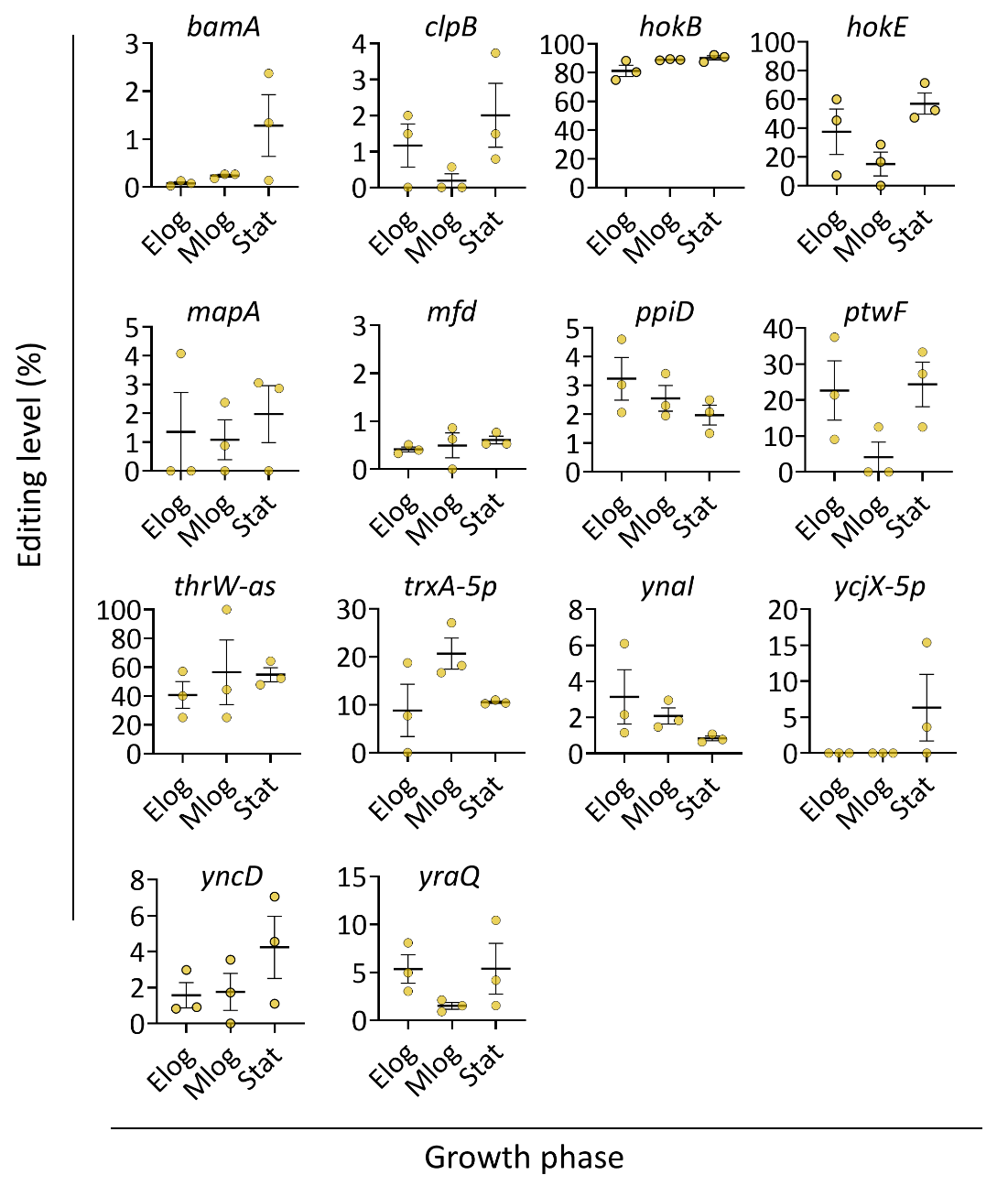
**

**Supplementary Figure 5. Editing levels of 14 mRNAs across growth phases when bacteria grew in LB.** Statistical analysis was conducted using one-way ANOVA with Tukey’s multiple testing correction. None of the sites showed a significant change across the different phases, but notice that in multiple cases, the average value at the stationary phase is higher, probably accounting for the significant difference in global editing levels observed between growth phases (Figure 2).

**
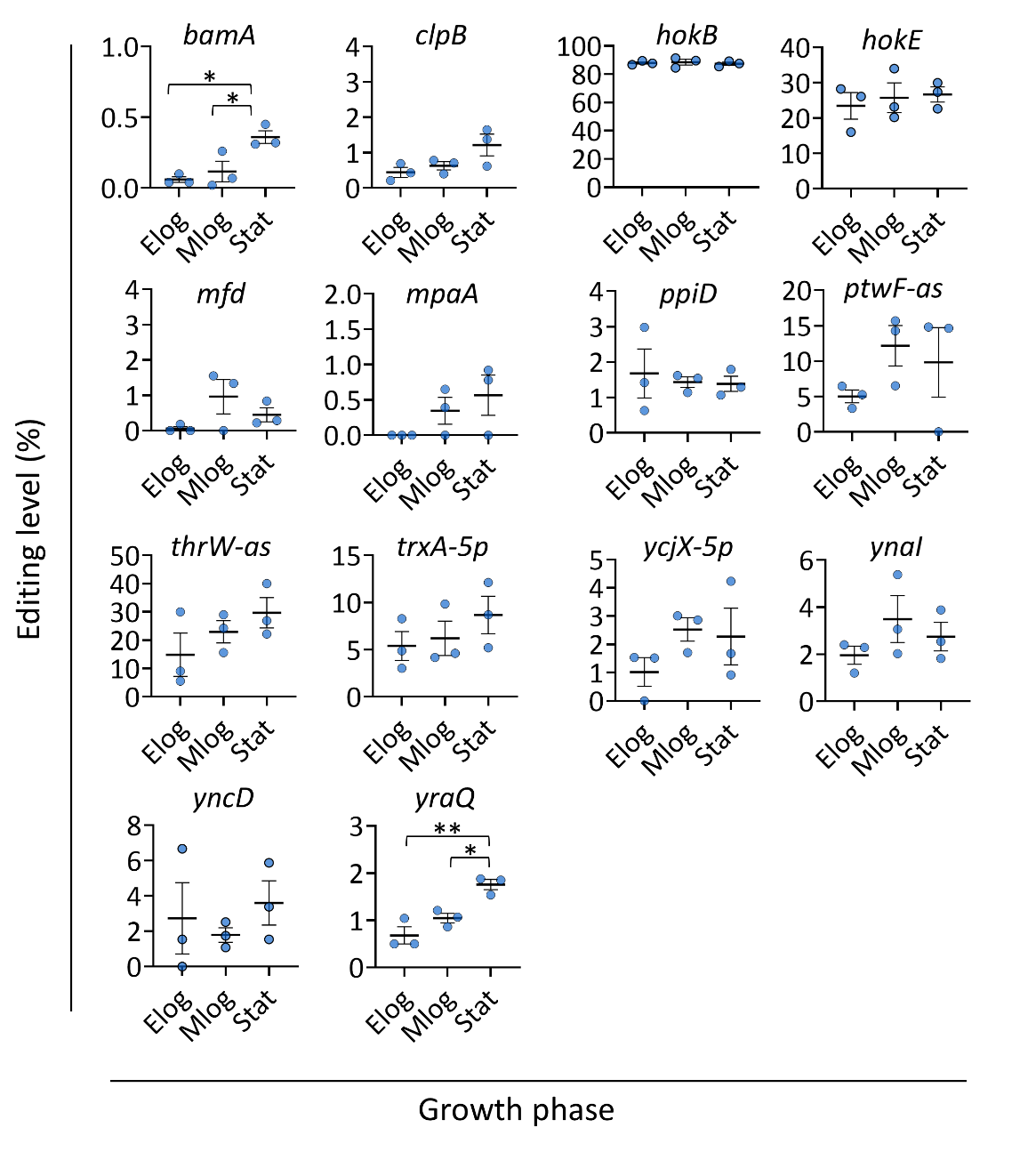
**

**Supplementary Figure 6. Editing levels of 14 mRNAs across growth phases when bacteria grew in M9.** Statistical analysis was conducted using one-way ANOVA with Tukey’s multiple testing correction. P-value marks are as follows: p ≤ 0.05 (*) and p ≤ 0.01 (**); only significant comparisons are marked.

**
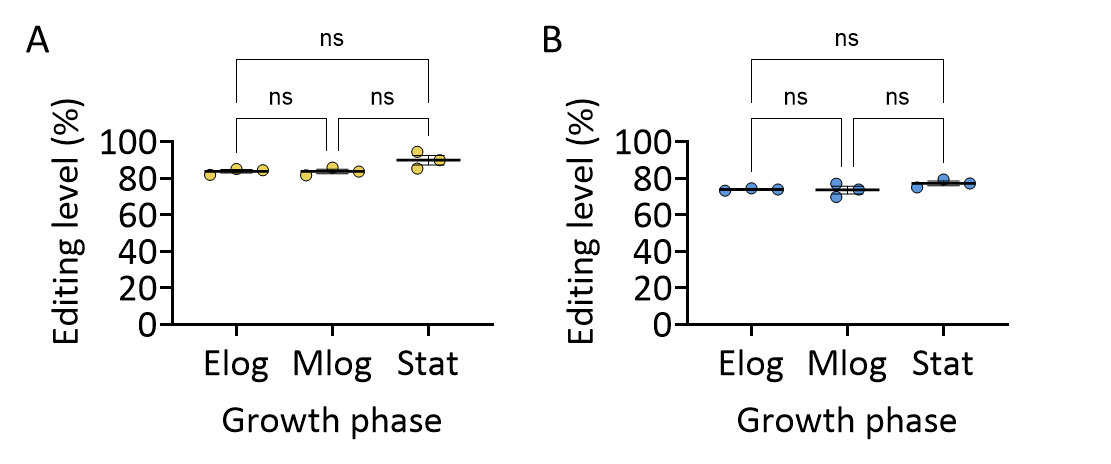
**

**Supplementary Figure 7. tRNA‑Arg2 editing levels do not change significantly across growth phases in bacteria grown in LB or M9.** tRNA-Arg2 editing level (averaged from editing levels of *argQ*, *argV*, *argY*, and *argZ*) as measured across growth phases in LB (left; yellow) and M9 (right; blue). Statistical analysis was conducted using one-way ANOVA with Tukey’s multiple testing correction. Values are found in Supplementary Table 5.

**
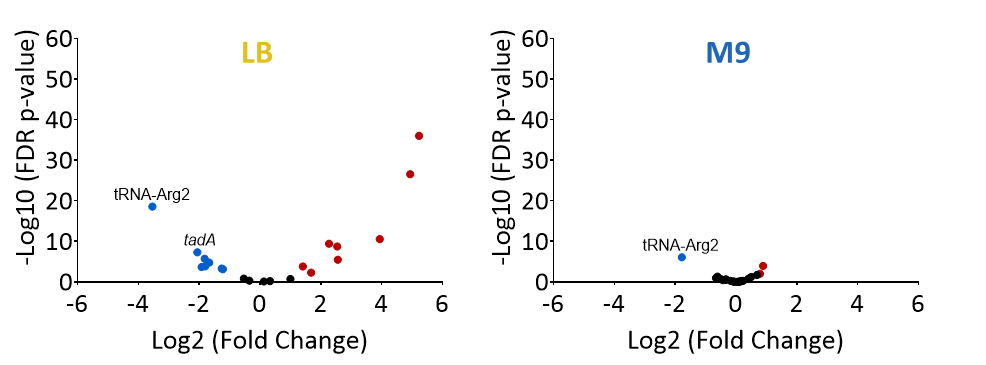
**

**Supplementary Figure 8. tRNA-Arg2 expression is significantly downregulated at the stationary phase.** Volcano plots showing the change in gene expression of 19 edited mRNAs, tRNA-Arg2, and *tadA* between samples in the Stationary phase and the mid-logarithmic phase in either LB or M9. Significant values (FDR p-value ≤ 0.01) are marked in blue and red for downregulated and upregulated genes, respectively.

**Supplementary Figure 9. tRNA-Arg2 editing levels are significantly higher in bacteria grown in LB than in M9 across all growth phases.** tRNA-Arg2 editing level (mean of the editing levels of *argQ*, *argV*, *argY*, and *argZ*) was measured across growth phases in LB (yellow) and M9 (blue). Statistical analysis was performed using one‑way ANOVA followed by Šídák’s multiple‑comparisons test. Significant differences are indicated as follows: p-value ≤ 0.01 (**); ≤ 0.001 (***).

**
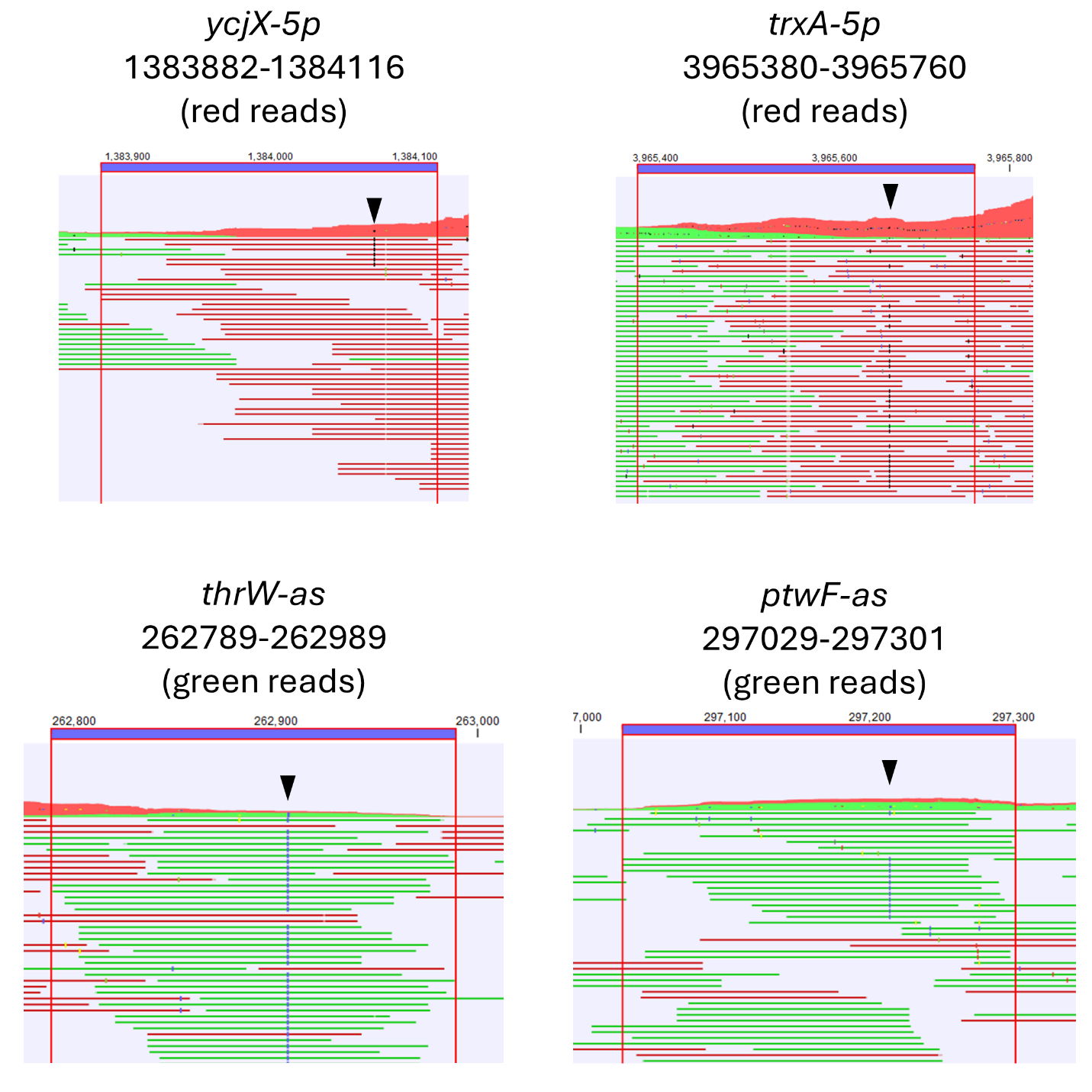
**

**Supplementary Figure 10. Added transcripts to the *E. coli* transcriptome for the RNA-seq differential analysis performed in the current work.** Screenshots of CLC genomic workbench genome browser of four edited sites (marked with a black arrow).

**Supplementary Tables**

**Supplementary Table 1. All A-to-G RNA-DNA mismatches that pass our filtering criteria were identified in LB and M9 at the early-log, mid-log, and stationary phases in the WT and TadA^m^.**

**Supplementary Table 2. Frequency of A-to-G sites within the UACG motif in the TadA^m^ strain, including sites that did not pass our filters.**

**Supplementary Table 3. Frequency of A-to-G sites within the UACG motif in the WT strain, including sites that did not pass our filters.**

**Supplementary Table 4. A-to-I mRNA edited sites and statistical analysis across growth media and phases.**

**Supplementary Table 5. A-to-I tRNA edited sites and statistical analysis across growth media and phases.**

**Supplementary Table 6. Differential gene expression analysis values used in Figure 3 and Supplementary Figure 9.**

**Supplementary Table 7. tRNA-Arg2 and *tadA* gene expression levels in the CtRNA+, CtRNA-, and PtRNA+ strains with respect to their control strains.**

**Supplementary Table 8. A-to-I RNA editing sites within the UACG motif in the WT (MAS1081) and CtRNA+ (TSS248) strains.**

**Supplementary Table 9. Statistical analysis of sites identified in Supplementary Table 8.**

**Supplementary Table 10. A-to-I RNA editing sites within the UACG motif in the Pempty (TSS50) and PtRNA+ (TSS253) strains.**

**Supplementary Table 11. Statistical analysis of sites identified in Supplementary Table 10.**

**Supplementary Table 12. A-to-I RNA editing sites within the UACG motif in the WT (MAS1081) and CtRNA- (MAS1100) strains.**

**Supplementary Table 13. Statistical analysis of sites identified in Supplementary Table 12.**

**Supplementary Table 14. TadA protein intensities as determined by protein mass spectrometry.**

**Supplementary Table 15. Differential gene expression and codon usage analysis of genes upregulated in LB vs M9 at the mid-log phase.**

**Supplementary Table 16. Mutations in the TadA^m^ strain compared to its parental WT strain, as identified by whole‑genome sequencing (DNA‑seq) of three biological replicates.**

**Supplementary Table 17. Primers used in this work.**
